# Performance Evaluation of an Integrated Fuzzy-Based Driving-Support System for Real-Time Risk Management in VANETs

**DOI:** 10.3390/s20226537

**Published:** 2020-11-16

**Authors:** Kevin Bylykbashi, Ermioni Qafzezi, Phudit Ampririt, Makoto Ikeda, Keita Matsuo, Leonard Barolli

**Affiliations:** 1 Graduate School of Engineering, Fukuoka Institute of Technology (FIT), 3-30-1 Wajiro-Higashi, Higashi-Ku, Fukuoka 811-0295, Japan; bd20101@bene.fit.ac.jp (E.Q.); mgm19201@bene.fit.ac.jp (P.A.); 2 Department of Information and Communication Engineering, Fukuoka Institute of Technology (FIT), 3-30-1 Wajiro-Higashi, Higashi-Ku, Fukuoka 811-0295, Japan; makoto.ikd@acm.org (M.I.); kt-matsuo@fit.ac.jp (K.M.); barolli@fit.ac.jp (L.B.)

**Keywords:** VANETs, IoT, WSN, SDN, Fog/Edge Computing, fuzzy logic, driver support system

## Abstract

The highly competitive and rapidly advancing autonomous vehicle race has been on for several years now, and it has made the driver-assistance systems a shadow of their former self. Nevertheless, automated vehicles have many obstacles on the way, and until we have them on the roads, promising solutions that can be achievable in the near future should be sought-after. Driving-support technologies have proven themselves to be effective in the battle against car crashes, and with Vehicular Ad hoc Networks (VANETs) supporting them, their efficiency is expected to rise steeply. In this work, we propose and implement a driving-support system which, on the one hand, could immensely benefit from major advancement of VANETs, but on the other hand can effectively be implemented as a stand-alone system. The proposed system consists of a non-intrusive integrated fuzzy-based system able to detect a risky situation in real time and alert the driver about the danger. It makes use of the information acquired from various in-car sensors as well as from communications with other vehicles and infrastructure to evaluate the condition of the considered parameters. The parameters include factors that affect the driver’s ability to drive, such as his/her current health condition and the inside environment in which he/she is driving, the vehicle speed, and factors related to the outside environment such as the weather and road condition. We show the effect of these parameters on the determination of the driving risk level through simulations and experiments and explain how these risk levels are translated into actions that can help the driver to manage certain risky situations, thus improving the driving safety.

## 1. Introduction

Road traffic accidents claim approximately 1.35 million lives each year and cause up to 50 million non-fatal injuries, with many of those injured people incurring a disability as a result of those injuries. And, the fact is, each of those deaths and injuries is totally preventable [1]. In this regard, industry, governmental institutions and academia researchers are conducting substantial research to provide proper systems and infrastructure for car accident prevention. The initiatives of many governments for a collaboration of such researchers has concluded to the establishment of Intelligent Transport Systems (ITSs). ITSs focus on the deployment of intelligent transportation technologies by combining cutting-edge information, communication, and control technologies to design sustainable information networks based on people, vehicles and roads.

As a main component of ITS, Vehicular Ad hoc Networks (VANETs) aim not only to save lives but also to improve the traffic mobility, increase efficiency, and promote travel convenience of drivers and passengers. In VANETs, network nodes (vehicles) are equipped with networking functions to exchange essential information such as safety messages and traffic/road information with one another via vehicle-to-vehicle (V2V), and with roadside units (RSUs) through vehicle-to-infrastructure (V2I) communications. Although VANETs are already implemented in reality introducing several applications, the current architectures face numerous challenges, thus are far from achieving full marks in safety.

To overcome the encountered challenges, the integration of many emerging technologies—Software Defined Networking (SDN), Cloud Computing, Edge/Fog Computing, 5G, Information- Centric Networking (ICN), Blockchain and so forth—within current VANETs is actively being proposed. Although each one of these emerging technologies promises to solve several issues, other approaches and technologies—Wireless Sensor Networks (WSNs), Internet of Things(IoT)—that have been around us for years, can be used as an effective complement to alleviate various limitations.

Alongside these technologies, various artificial intelligence approaches including fuzzy logic and machine learning, are paving the way not only for a complete deployment of VANETs but also for reaching a bigger goal, that of putting the fully autonomous vehicles on the roads. Nevertheless, fully driverless cars still have a long way to go and the current advances fall only between the Level 2 and 3 of the Society of Automotive Engineers (SAE) levels [2]. Until we have self-driving vehicles, many automotive companies and academia researchers will continue working on Driver-Assistance Systems (DASs) as a principal safety feature to enhance the driving safety in non-automated vehicles. These intelligent systems reside inside the vehicle and rely on the measurement and perception of the surrounding environment, and based on the acquired information, a variety of actions is taken to ease the driving operation.

**Figure 1:**
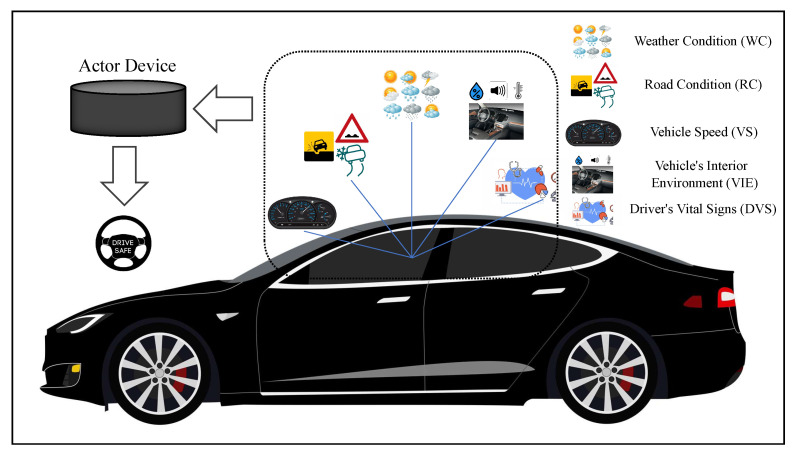
A visualization of the proposed system architecture.

Apart from the external factors, there are other factors that have an effect on the driving operation as well. The first and the foremost is the driver itself, as the driver’s ability to drive is rigorously related with the driving operation. By considering these important factors, we present and implement a non-complex and non-intrusive intelligent driving-support system to detect a danger or a risky situation in real time and warn the driver about the danger. Our system is based on fuzzy logic and makes use of the information acquired from various in-car sensors as well as from communications with other vehicles and infrastructure to evaluate the condition of the considered parameters. The parameters include factors that affect the driver’s ability to drive, such as his/her current health condition and the inside environment in which he/she is driving, the vehicle speed, and factors related to the outside environment such as the weather and road condition. A model of the proposed system is presented in Figure 1. We evaluate the proposed system by computer simulation and implement a testbed to attain experimental results. For the implementation of the testbed, we use some IoT devices equipped with various sensors from which we obtain the driver’s vital signs and data regarding the inside environment.

The remainder of this paper is as follows. Section 2, gives a background overview of the emerging technologies integrated within VANETs which enable the full implementation of our proposed system, as well as a short review of several research papers relevant to this work. The proposed fuzzy-based driving-support system and the details of its implementation are presented in Section 3. Section 4, discusses the simulation and experimental results. The last section, Section 5, gives some concluding remarks and ideas for the future work.

## 2. Background Overview and Related Work

In this section, we present a brief introduction of IoT, WSNs, Cloud, Fog and Edge computing, and SDN as promising technologies for full implementation and management of VANET applications. In addition, we give a literature review of several pieces of research related to this work.

### 2.1. Internet of Things

In general, the IoT is a networking concept that refers to the rapidly growing number of devices able to communicate and interact with others over the different types of networks with the aim of creating a smart environment which will add more ease to daily life for the people worldwide. Although there are many components involved in IoT, ITSs and therefore VANETs are an essential part when it comes to the development of one of the most important IoT applications—Smart Cities. ITSs include intelligent systems which help to better manage traffic, cut pollution, make better use of infrastructure and help citizens stay safe and clean. However, most ITSs rely on expensive infrastructure, and alternatives which reduce the required investment are to be sought-after.

### 2.2. Wireless Sensor Networks

WSNs have become more mature over the years and will continue to give momentum to many applications for the features it provides. As one of the technologies that take part in ITS, WSNs are seen as key components of heterogeneous systems cooperating along with other technologies employed in vehicular scenarios, especially due to the little installation and maintenance costs [3]. They can be deployed along urban roads and highways, intersections and in parking areas to constantly obtain information about the weather and road condition, the traffic state and so forth.

### 2.3. Cloud, Fog and Edge Computing

Fog computing is a highly virtualized platform that has many features that make it an appropriate platform to deliver numerous services in safety, traffic support, infotainment, and analytics: geographical distribution (across cities, and along roads and highways), low latency, mobility support and location awareness among others [4]. However, despite its great attributes, it does not totally exclude the necessity of Cloud computing in VANETs. Given the significant increase of the amount of data generated by the ever increasing number of sensors, devices, and more importantly, vehicles; Cloud computing will continue to have its crucial role in VANETs as it provides the necessary resources for big data management and analytics.

Edge computing in VANETs, or the so-called Vehicular Cloud Computing (VCC), Volunteer Computing-based VANET (VCBV), Vehicular Edge Computing (VEC) or whatever this technology is named as it is roughly the same thing, consists of vehicles equipped with resources and services of networking, computing, storage and control capabilities which are used to process a considerably amount of data at/through the vehicles, therefore offloading massive traffic flows from core networks. If that data is sent back across a long network link to be analyzed, logged and tracked, that takes much more time than if the data is processed at the edge, close to the source of the data [5].

### 2.4. Software Defined Networking

Software Defined Networking (SDN) is a prospective solution in managing complex networks with little cost while providing optimal resource use. SDN offers a scalable and programmable network architecture which simplifies the network planning and design, given its global knowledge of the network. In VANETs, SDN enables the deployment of different applications simultaneously while offering flexibility and adaptation to the rapid changes of the network. It does so by prioritizing the changing applications’ requirements and conditions in delay, propagation and bandwidth; changes which can be very effective in particular cases (e.g., emergency cases) [6,7].

### 2.5. Vehicular Ad Hoc Networks

**Figure 2:**
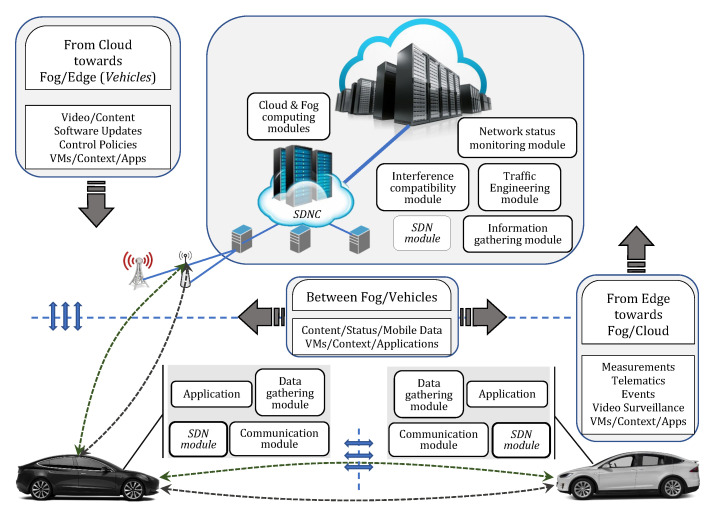
Cloud-Fog-Edge Computing and SDN integrated within VANETs and the content flow in this novel architecture.

By integrating all the aforementioned technologies within VANETs, a hybrid system between centralized and distributed computing/network-control is created [8]. The basic components of this network architecture with the content distribution is given in Figure 2.

Certainly, vehicles constitute the key component of this integrated architecture as most of the information that flows in the network will pass through them at some point, i.e., vehicles can be at the same time a source of information, a relay node or a destination point of the information, which, on the other hand, can be comprised of only raw data, only processed or both of them. In addition, although different types of sources are used to acquire the same type of information, different types of information serve the same destination/application. Nonetheless, the other components—data centers, fog servers, SDN Controllers (SDNC), 5G base stations, RSUs and RSU Controllers (RSUCs)—are almost as important as vehicles, because the traditional VANETs architecture which was based only on the vehicles, has failed to meet the demands.

The sensors installed along the roads monitor the driving environment (e.g., traffic density, weather condition, the type and condition of the road) and send the sensed data to the nearest RSU. In this process, the vehicles moving nearby can also help by working as relay/processing nodes, so the sensors do not waste energy to send the generated data farther. Vehicles, which also monitor the driving environment through their sensors and cameras, can help in the processing operation if they possess the required resources. Otherwise, Cloud or Fog resources will do all the processing tasks depending on the application requirements. For instance, if the processing is needed in real time, the resources to be used are those of Fog. Cloud servers are used as a repository for software updates, control policies and for the data that need long-term analytics and are not delay-sensitive. SDNC does, by all means, the orchestration of all the processes and components involved in this architecture [9].

The implementation of this architecture promises to provide numerous services for drivers and passengers and will set the scene for future autonomous and connected cars.

### 2.6. Related Works

The highly competitive, and rapidly advancing, autonomous vehicle race has been on for several years now and it is a matter of time until we have these vehicles on the roads. However, even if the automotive companies do all what it takes on their end to create the fully automated cars, there will still be one big obstacle, the infrastructure. In addition, this could take decades even in the most developed countries. Moreover, 93% of the world’s fatalities on the roads occur in low- and middle-income countries [1] and considering all these facts, DASs should remain the focus of interest for the foreseeable future.

DASs can be very helpful in many situations as they do not depend on the infrastructure as much as the driverless vehicles do. Furthermore, DASs can provide driving-support with very little cost, thus help in low- and middle-income countries in the long battle against car accidents. Most of these systems concentrate on maintaining driver’s attention and intervene when he/she seems incapable of driving safely. There are different ways to detect the driver inattention: by monitoring the driver directly, by analyzing the driving performance and a way which combines the first two.

DASs have been in the focus of many researchers of the automotive industry and academia for many years and their work has been paying off given the continuous improvement of such systems. In [10], authors review several projects executed by some major automotive companies such as Toyota, Nissan, BMW, Mercedes-Benz, Saab, and Volvo, and by companies such as Veoneer, Seeing Machines and SmartEye which are specialized only at the research and development of these systems. These projects have the driver inattention (coming from fatigue, drowsiness and distractions) in their focus, for which they use smart cameras to track several facial features and then process the acquired data using computer vision algorithms. There are also many researchers from academia that are engaged in the development of DASs. In [11], authors implement a vision system for monitoring driver’s vigilance by integrating the facial features of the eyes, mouth and head. In et al. [12], authors deal with driver fatigue for which they propose a probabilistic model by considering head, eyelid and gaze movement along with driver’s facial expressions. There are also many other research works that use such behavioral measures to detect driver inattention. These include percentage of eyelid closure (PERCLOS) [13,14], eye blinking [13,15,16], pupils’ motion [17], yawning [18], as well as other facial expressions—jaw drop, lip/mouth stretch, inner/outer brow raise, and such [19,20]. The authors communicate interesting results and good performance of their systems. However, since they use facial feature extraction and vision-based approaches, their systems encounter many limitations. Vision-based approaches require a long moving-averaged window to track slow changes in the driver’s vigilance. Furthermore, they experience several illumination issues, especially during night-times. Infrared illumination techniques can be very effective in such cases [13,21,22], but in a case of light reflection (sunlight or reflections from glasses), the performance declines by 30% [13].

Other researchers use driver’s biological signals e.g., *electrocardiogram* (ECG), *electroencephalogram* (EEG), *electromyogram* (EMG) and *electro-oculogram* (EOG), to detect his/her drowsiness and report that these biopotentials can be used to detect drowsiness with a high accuracy [10,23]. Heart rate also varies considerably between the different stages of drowsiness, such as alertness and fatigue [24,25]. In addition to heart rate, respiratory rate can be used as an indicator of mental stress [26].

Driving performance measures are proven to be an effective way of detecting driver inattention as well, since changes in the mental state while driving are reflected in the driving performance. To detect driver fatigue, Zhong et al. [27] performed a localized energy analysis on the vehicle state information, such as steering angle and trace profile. They conclude that the localized energy changes with the driver’s mental state. In [28], authors estimate driver fatigue through steering motion. They apply chaos theory to explain the change of steering-wheel motion, and by analyzing the chaos characteristics they can detect signs of possible fatigue episodes. Other research works consider different parameters including the steering-wheel position, accelerator pedal position, forces on the pedals, lane boundaries, upcoming road curvature, and vehicle velocity [29,30,31,32]. The aforementioned research works detect the driver inattention based on driver-vehicle interaction, which can be effective in many cases, yet it requires modifications of the vehicle structure, which is inappropriate in a real assessment [33].

On the other hand, there are several research papers that are in line with our research work regarding the method used to determine the driver’s situation. In [13], authors use PERCLOS, eye closure duration, blink frequency, fixed gaze, frontal face pose and nodding frequency as input parameters to a fuzzy inference system. They reported that their system detects driver fatigue with 98% accuracy. Nevertheless, the performance of the system is severely affected by the light reflections which cause a 30% decline in detection accuracy. A more straightforward fuzzy system considering the following inputs: head-nodding frequency, posture adjustment frequency, slouching frequency and PERCLOS is implemented in [34]. Even though authors consider less input parameters for the implementation of the system, they report that it runs slow and state that an implementation of the system using C language in place of Matlab could facilitate it to run much faster. A thorough work in regard to the diversity of considered inputs is presented in [33]. Vehicle speed, PERCLOS, heart rate, blood pressure and temperature are the parameters that authors consider for their driver monitoring system. They design a Fuzzy Bayesian framework which analyzes the condition of the considered variables and then determines whether the driver can drive safely. Although authors report a good resolution and flexibility of the system, they also state a high computational complexity which is determined by the number of fuzzy members in each membership function and by the number of nodes in the Bayesian network.

## 3. Proposed System

Although most of the above-mentioned research works focus on driver inattention, it is noteworthy to also consider other factors which often are determined to be a leading cause of many accidents: the road and weather condition. In many cases, these two parameters are a determinant factor alone, but a combination with an inappropriate speed and with a driver who is experiencing both mental and physical discomfort, would lead the driver to a fatal situation. Therefore, the objective of our work is to develop a non-complex and non-intrusive intelligent driving-support system that can detect a dangerous situation in real time by taking into consideration different types of parameters that affect the driving process.

We use fuzzy logic to implement the proposed system because the situation we want to deal with involves many uncorrelated parameters, which in turn lead to a non-deterministic polynomial-time hard (NP-hard) problem. Different approaches can be used to deal with NP-hard problems, but fuzzy logic is the most efficient in solving decision making and control problems in real time. In addition, the fuzzy rule expression is close to an expert natural language, thus it allows the modeling of such inherently ambiguous notions as driving situations in an efficient and effective way [35,36,37,38,39,40].

### 3.1. Proposed Fuzzy-Based System Description

**Figure 3:**
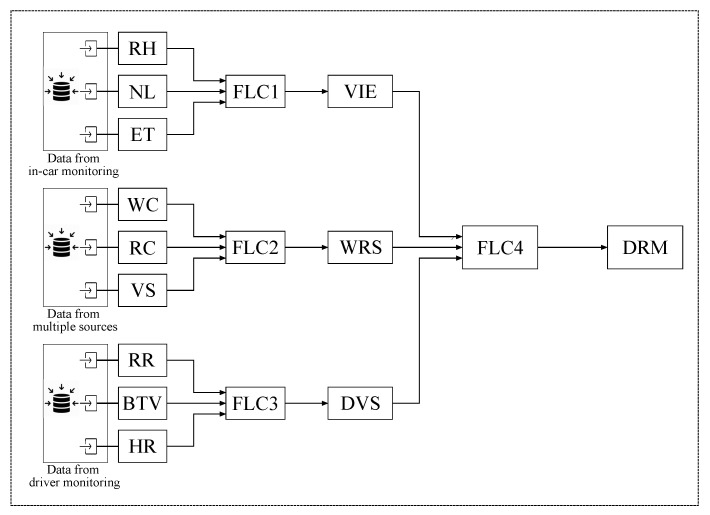
A diagram of the proposed fuzzy-based system.

The proposed system is shown in Figure 3. It consists of four Fuzzy Logic Controllers (FLCs) and nine input parameters. Although it seems more complex to use four FLCs, it is far better than having nine input parameters in a single FLC because this would result in a very complex Fuzzy Rule Base (FRB) composed of thousands of rules, which, in turn, would increase the overall complexity of the system. The considered parameters are explained in the following.

*Relative Humidity (RH)*: Humidity is one of the air quality parameters that considerably affects the driving comfort and human health [41]. The driver and passengers inside the vehicle continuously generate moisture which results in an increase of the relative humidity. Contrarily, the air-conditioner which produces hot, dry air, decreases the RH if it is left on. A very high or very low RH both cause great discomfort, which can make the driver not to focus only on the driving process.

*Noise Level (NL)*: Ambient noise impacts driver’s mental state by increasing general stress levels and aggravating stress-related conditions. Traffic noise and vehicle noise (the noise generated by the vehicle itself), generally speaking, are the main sources of noise which most of the drivers suffer from. A stressed driver, is consequently, a source of dangerous situations; therefore, we consider this parameter in our system.

*Environment Temperature (ET)*: Temperature is another environmental factor which is considered to be able to affect the driver as well. In [42], authors show that temperature variations can be used to combat drivers’ drowsiness. The results indicate that the likelihood of drowsy driving is significantly reduced by maintaining a cooler inside temperature in the vehicle.

*Weather Condition (WC)*: According to Federal Highway Administration, approximately 21% of car crashes that happen every year are weather-related. Weather acts through visibility impairments, precipitation, high winds, and temperature extremes to affect driver capabilities and vehicle performance (i.e., traction, stability and maneuverability) [43].

*Road Condition (RC)*: Although the weather and road condition are closely related, it should be considered to be a separated parameter since a change of weather conditions is not always reflected in a change of road conditions. For instance, after a heavy rain or snow even if the weather gets better, the roads might still be slippery for a while, or a flooding may happen, which in turn could damage the roads. Moreover, the roads may have potholes, or they may be bumpy, and such poor road conditions are not related with the current weather condition. The authors in [44], present a system which monitors the road condition in real time.

*Vehicle Speed (VS)*: The speed at which a vehicle moves has a direct impact on a car crash as well as on the severity of injuries resulting from that crash. Specifically, an increase of 1 km/h in average speed typically results in a 3% higher risk of a crash involving injury, with a 4–5% increase for crashes that result in casualties [1]. In addition, if the speed is combined with a bad condition of the other parameters, the impact certainly escalates.

*Respiratory Rate (RR)*: The respiratory system experience significant changes from wakefulness to drowsiness, thus it makes the breathing rate an interesting variable which can be used to detect driver’s drowsiness. Its high degree of effectiveness is reported in various studies [45]. In addition to drowsiness detection, RR can be used to detect mental stress which is in our interest as well.

*Body Temperature Variation (BTV)*: Effects of the increase of body temperature range from discomfort (feeling hot, sweating, feeling thirsty, slightly hungry etc.) to fainting, dizziness weakness, headache and more, which could lead to fatal driving situations. On the other hand, since the body temperature drops during sleep, it can be used to detect whether the driver is awake or falling asleep.

*Heart Rate (HR)*: As with RR, HR is one of variables that can detect drowsiness as it varies considerably between the different stages of drowsiness, such as alertness and fatigue [24,25]. Furthermore, if the driver is experiencing anxiety or stress, his/her heart rate will increase, thus we can use it to determine different situations which could influence the risk of a crash.

Vehicle Inside Environment (VIE), Weather-Road-Speed (WRS) and Driver’s Vital Signs (DVS) are the output variables of FLC1, FLC2 and FLC3, respectively, and at the same time, serve as input parameters for FLC4. The final output of our system is Driving Risk Management (DRM), which determines the degree of risk that a situation involves at the moment.

The term sets of used linguistic parameters are defined respectively as:

$$\begin{array}{ccc} T(RH) & = & \{Low\;(L),\;Medium\;(M),\;High\;(H)\};\\ T(NL) & = & \{Quiet\;(Q),\;Noisy\;(N),\;Very\;Noisy\;(VN)\};\\ T(ET) & = & \{Low\;(Lo),\;Medium\;(Me),\;High\;(Hi)\};\\ T(WC) & = & \{Very\;Bad\;(VB),\;Bad\;(B),\;Good\;(G)\};\\ T(RC) & = & \{Very\;Bad\;(VBa),\;Bad\;(Ba),\;Good\;(Go)\};\\ T(VS) & = & \{Slow\;(Sl),\;Moderate\;(Mo),\;Fast\;(Fa)\};\\ T(RR) & = & \{Slow\;(Sl),\;Normal\;(Nm),\;Fast\;(Fs)\}.\\ T(BTV) & = & \{Small\;(Sm),\;High\;(H),\;Very\;High\;(VH)\};\\ T(HR) & = & \{Slow\;(S),\;Normal\;(No),\;Fast\;(Fa)\};\\ T(VIE) & = & \{Extremely\;Uncomfortable\;(EUC),\;Very\;Uncomfortable\;(VUC),\\ & & \;Uncomfortable\;(UC),\;Moderate\;(Mod),\;Comfortable\;(C)\};\\ T(WRS) & = & \left\{No/Minor\;Danger\;(N/MD),\;Moderate\;Danger(MD),\;Considerable\;Danger(CD\right)\\ & & \;High\;Danger(HD),\;Very\;High\;Danger(VHD)\};\\ T(DVS) & = & \{Extremely\;Bad\;(EB),\;Very\;Bad\;(VB),\;Bad\;(B),\;Fair\;(F),\;Good\;(G)\};\\ T(DRM) & = & \{Safe\;(Sf),\;Very\;Low\;(VL),\;Low\;(Lw),\;Moderate\;(Md),\;Considerable\;(Co),\\ & & \;High\;(Hg),\;Very\;High\;(VH),\;Severe\;(Sv),\;Danger\;(D)\}. \end{array}$$

Based on the description of linguistic parameters we make the Fuzzy Rule Base (FRB). The FRB forms a fuzzy set of dimensions $|T\left(x_{1}\right)|\;\times \;|T\left(x_{2}\right)|\;\times \;\cdots \;\times \;|T\left(x_{n}\right)|$, where $|T(x_{i})|$ is the number of terms on $T(x_{i})$ and *n* is the number of input parameters.

FLC1, FLC2 and FLC3 all have three input parameters with three linguistic terms each, therefore, there are 27 rules in each FRB. The FRB of FLC 4 has 125 rules because it consists of three input parameters with every input having five linguistic terms. FRB1, FRB2, FRB3 and FRB4 are shown in Table 1, Table 2, Table 3 and Table 4, respectively. The rules of FRB are IF-THEN statements which are used in the decision process when an actual input is given. For instance, FRB 1, Rule 1: “IF RH is L, NL is Q and ET is Lo THEN VIE is UC” or for FRB4, Rule 100: “IF VIE is Mod, WRS is VHD and DVS is G THEN DRM is Co”.

**Figure 4:**
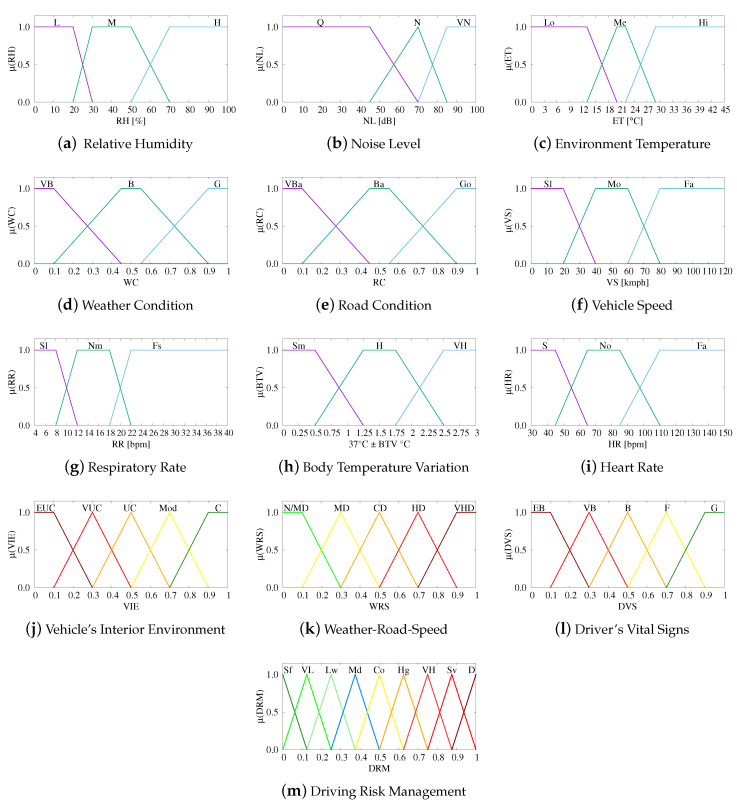
Membership functions of input and output parameters.

To maintain input-output continuity, an important feature that recommends that small changes of input parameters should result in small changes of output values, we use five membership function for the outputs of FLC1, FLC2 and FLC3 (VIE, WRS and DVS, respectively) and nine for the output of FLC4 (DRM). This way we avoid too many control rules to fall into the same decision level, thus a better continuity is achieved. The membership functions used for all parameters are given in Figure 4. We use triangular and trapezoidal membership functions because they are suitable for real-time operation. We decided the numeric range of each membership function first by following the recommendations and guidelines for humidity, indoor temperature, noise levels etc., and then we adjusted them during the design process which included many computer simulations.

### 3.2. Testbed Description

**Figure 5:**
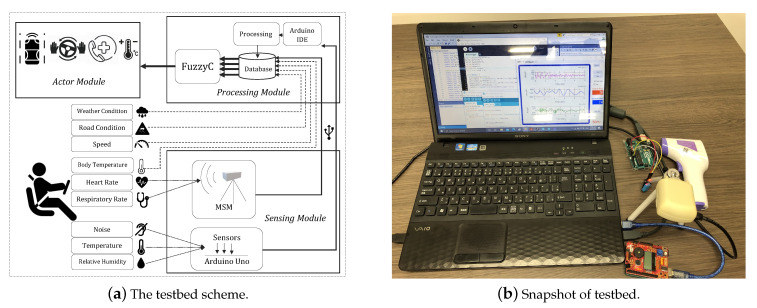
A scheme and a snapshot of the implemented testbed.

With the purpose of testing the proposed system in a real scenario, we designed and implemented a portable and non-intrusive testbed which allowed us to acquire indispensable data for our system. The design and a snapshot of the implemented testbed is given in Figure 5. The testbed includes the following: an automated sensing module, a manual data input, a processing module and an actor module instancing different actions that could be carried out when the system’s output (DRM) exceeds certain values.

The automated sensing module consists of several sensors setup on an Arduino Uno used to measure the relative humidity, temperature and noise in the vehicle’s interior environment and a DC6M4JN3000 Microwave Sensor Module (MSM) used to get the driver’s heart and breathing rate. Although the data for these parameters is transferred to the processing module via USB cables, the data of body temperature, speed and weather/road condition parameters are entered directly into the processing module database through a manual data input.

The processing module implemented in Windows OS, first makes use of Arduino IDE and Processing software to obtain and convert the parameters data into readable data for our Fuzzy program i.e., FuzzyC in which we implement our system, and then it runs the FuzzyC through Visual Studio software to determine the DRM value. Afterwards, the actor module decides the relevant action to be taken based on the degree of risk which is induced by the DRM.

## 4. Proposed System Evaluation

This section presents the simulation results of the proposed system together with the experimental results from the implementation in a real scenario. We describe in detail the relation between driving risk levels and the input variables, and explain how these risk levels are translated into actions that can help the driver to manage certain risky situations with the goal of improving the driving safety.

### 4.1. Simulation Results

**Figure 6:**
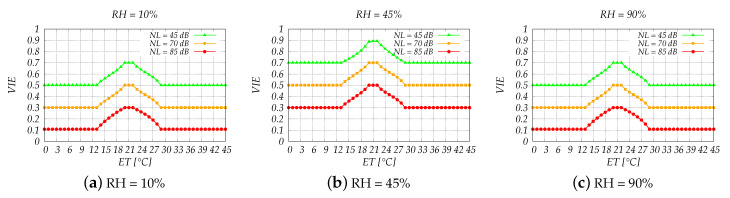
Simulation results of FLC1 for low, medium and high humidity.

**Figure 7:**
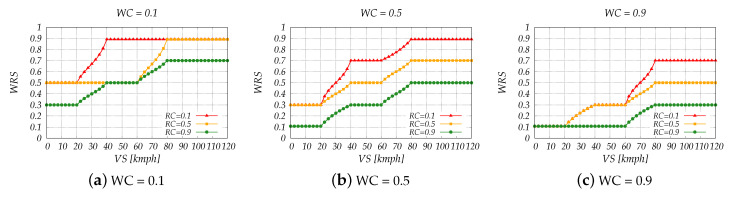
Simulation results of FLC2 for very bad, bad and good weather condition.

**Figure 8:**
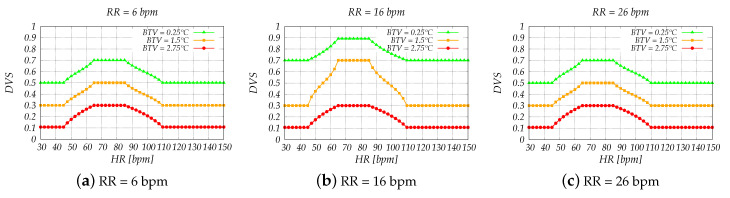
Simulation results of FLC3 for slow, normal and fast respiratory rate.

**Figure 9:**
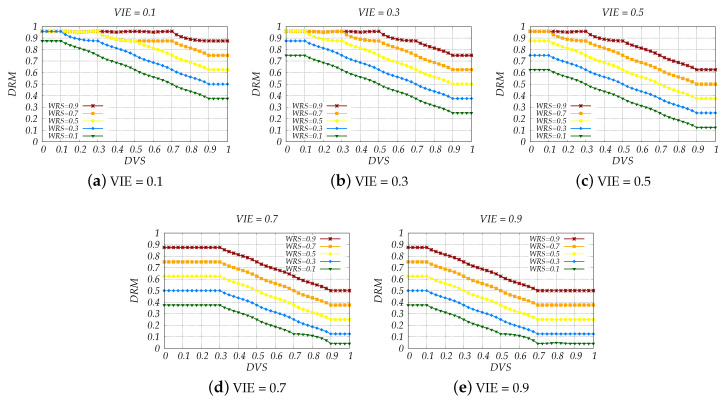
Simulation results of FLC4 for extremely uncomfortable, very uncomfortable, uncomfortable, moderate and comfortable vehicle inside environment.

Grouping together the variables not only decreased the overall complexity of the system but also helped us to better understand the results and single out implementation flaws. The simulation results are shown in Figure 6, Figure 7, Figure 8 and Figure 9.

In Figure 6 is shown the relation between VIE and ET for different RH and NL values. Regarding RH, we consider the scenarios with a dry, normal and humid inside environment represented by a 10%, 45% and 90% relative humidity, whereas for the NL parameter, we consider the values 45, 70 and 85 dB which simulate a quiet, noisy and very noisy environment, respectively.

Relative humidity plays an important role in the comfort levels and we can see this fact also in our results. When the inside ambient is dry or humid (see Figure 6a,c), we can see that there is not any situation that the vehicle inside environment is decided as comfortable. As you can see from Figure 6b, the only scenario when the environment is comfortable (VIE = 0.9) is when the humidity is in normal levels. However, regardless the normal levels of RH, if driver is driving in low/high temperatures or in a noisy environment, VIE is never decided as comfortable. Anyway, a moderate level of comfort is still acceptable, but any other level may have an impact on the driver, which in turn may affect the driving performance.

In Figure 7 is shown the relation between WRS and VS for different WC and RC values. The values 0.1, 0.5 and 0.9 of both WC and RC, simulate a very bad, bad and good weather/road condition, respectively.

Figure 7a shows the considered case with the worst weather condition (WC = 0.1). As expected, most of WRS values show a very high danger. When it is combined with a very bad road condition (RC = 0.1), the scenario with the lowest degree of danger is when the vehicle is moving slowly, and yet is decided as a situation with a Considerable Danger (WRS = 0.5). The increase of RC—better road conditions—results in the decrease WRS values. The WRS values are decreased even more when the weather gets better (see Figure 7b,c) and when both the weather and road condition are good (WC = 0.9 and RC = 0.9), many situations are decided with No or Minor Danger—WRS = 0.1. Driving at a high speed poses a threat itself, regardless the road or weather condition; therefore, these scenarios are not decided with a “No/Minor Danger” on any occasion.

The effect of HR, RR and BTV in the determination of DVS values is shown in Figure 8. For the simulations, we consider a RR of 6, 16 and 26 bpm, and a BTV of 0.25, 1.5 and 2.75 °C. The considered HR values range from 30 to 150 bpm.

A good condition is determined only when all three variables are within the normal range (Figure 8b, for BTV = 0.25 °C and HR between 60 and 95 bpm). If one of the vital signs deteriorates, DVS values immediately drop to the fair level (DVS = 0.7) and when it happens to two of them, DVS values decrease even more. Thus, the system can detect if the driver is experiencing anxiety and stress since the heart and breathing rate speed up in these situations. The opposite happens when the driver is drowsy, his/her heart and respiratory rate decreases, yet again the system can detect it. Moreover, in these situations the body temperature also drops, thus helping to determine such cases with a better accuracy.

In Figure 9, we give the relation between DRM and DVS while considering all the possible levels of VIE and WRS. In other words, we consider the values 0.1, 0.3, 0.5, 0.7 and 0.9 which imply scenarios with an extremely uncomfortable, very uncomfortable, uncomfortable, moderate and comfortable environment, respectively. However, for WRS, we consider No/Minor, Moderate, Considerable, High and Very High Danger simulated through the values of 0.1, 0.3, 0.5, 0.7 and 0.9.

In Figure 9a, we consider the VIE value 0.1 and change the WRS from 0.1 to 0.9. We can see that all DRM values are greater than 0.3125 (the border between low and moderate risk) which imply the existence of risky situations. This is due to extremely uncomfortable VIE i.e., dry/humid, cold/hot and very noisy environment, as it could affect the driver’s ability to focus on the driving process. In addition, if the road and weather condition are not good, or the driver is driving at a high speed, the risk levels are decided even up to the “Danger” level—DRM ≥ 0.9375. If driver’s vital signs indicate a very good condition of his/her physical and mental health, we can see a slight improvement on the risk levels, yet not enough for the situation to be decided with a low risk.

By comparing the DRM values for all the considered cases, we can see the great impact of the input parameters on the determination of the driving risk. As we can see from Figure 9c–e, when VIE is equal or greater than 0.5, situations with very low risk are now present and happen when the driver is in good health condition while WRS remains low. Regarding WRS, a driver with a good mental and physical condition can manage to drive a comfortable vehicle with a low risk only if WRS remains below 0.5.

If several consecutive DRM values are above the low risk level, the system can invoke a certain action. For instance, when the DRM values fall within the moderate level, the system could take action to lift the driver’s mood or improve the vehicle inside environment. If the risk is caused by a very low DVS, which means that the driver is in a very bad condition, the system should act immediately. For example, the system suggests or urges the driver to pull over and take a rest based on the degree of DVS, and at the same time reports his/her health status to the doctor. When the DRM values are very high, the system could even decide to lower the speed limit to a speed that the risk is reduced significantly.

### 4.2. Experiment Setup and Results

At this time, we have implemented and run the proposed system only in one case scenario. The experiment was made to test and see the response of the system in a real scenario rather than to determine its accuracy.

**Figure 10:**
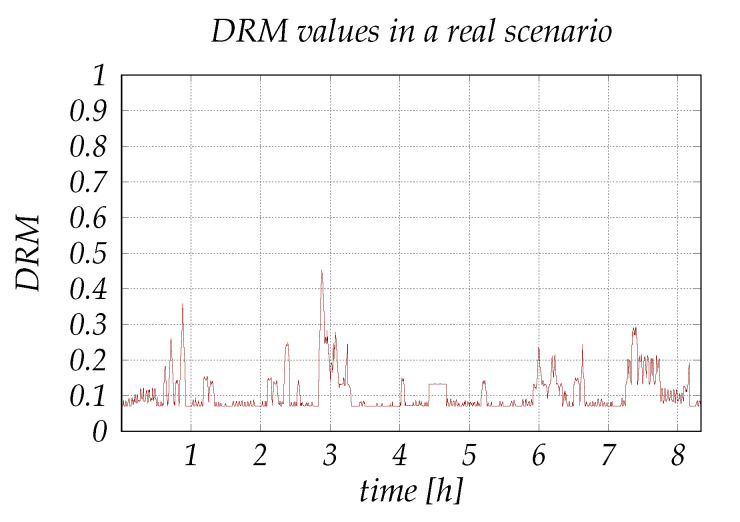
Experimental results.

The results shown in Figure 10, present the DRM values determined by the system using the data collected during approximately 8 h of driving, in an opportunistic process which took place between 6 a.m. and 11 p.m., mostly in urban driving environment. Considering the nature of the input parameters—different parameters change differently over time and with different frequencies i.e., speed and noise may change instantaneously but humidity and temperature change gradually—we set the system to decide an output every 15 s. This was made to also permit the manual data input (the automated sensing module runs in real time) and to minimize the error of the automated part by calculating the average value of the samples collected during the 15-s time frame.

The results indicate that most of the driving situations are determined with no risk present, with several situations showing risks to certain degrees. The low DRM values are an outcome of the driving scenario which took place mostly in urban environment and during a day with a good weather, with the driver being in a good mental and physical condition, while the small fluctuations are caused by the frequent changes in the noise levels and in speed due to presence of traffic lights, crossroads and pedestrians. To obtain a variation in the condition of some parameters, we adjusted the heating and cooling functions (in some cases it was turned off) so the temperature and humidity could change, varied the sound volume produced by the vehicle audio and also drove to some countryside areas. As it appears, the DRM values fluctuate between different risk levels, and the cases with higher DRM values are the scenarios that include the combination of several parameters with bad/fair conditions.

## 5. Conclusions

In this paper, we gave a brief overview of emerging technologies like IoT, WSNs, Edge/Fog computing and SDN as technologies which can be integrated within VANETs to achieve a full deployment and management of vehicular applications and services. Moreover, we showed how these technologies can help the driving-support systems and especially how the system that we propose through this work can benefit from them.

The proposed system which is implemented using fuzzy logic determines the driving risk in real time and provides an output that can be used to take different actions when the risk exceeds certain limits. By performing the appropriate actions, the risk can be reduced, thus increasing driving safety. For its implementation we considered nine input parameters: the vehicle’s inside environment temperature (ET) and relative humidity (RH), noise level (NL), the driver’s vital signs i.e., heart rate (HR), respiratory rate (RR) and body temperature variation (BTV), the road condition (RC), weather condition (WC) and the vehicle speed (VS). The effect of these parameters on the determination of the driving risk was showed through simulations and experiments.

Nevertheless, since our experiment did not include any sort of accuracy evaluation, it is noteworthy to determine its accuracy as it can show which parameters lead mostly to a false positive output and what should be improved to reduce the false negative outputs. Therefore, we intend to evaluate the performance by looking into correct detection in many realistic case scenarios which would involve different weather conditions, several drivers and more driving environments.

In the future, we would like to further improve our integrated system by switching the parameters that lead to false positives with others that are a cause of many accidents, with the vehicle technical condition being one of them, as well as with parameters that determine accurately the driver’s ability to drive, e.g., EEG and PERCLOS; to achieve what is ultimately the goal of the driving-support technologies, reducing the risk of car accidents.

## Figures and Tables

**Table 1:** Fuzzy Rule Base of FLC1.

No.	RH	NL	ET	VIE	No.	RH	NL	ET	VIE	No.	RH	NL	ET	VIE
1	L	Q	Lo	UC	10	M	Q	Lo	Mod	19	H	Q	Lo	UC
2	L	Q	Me	Mod	11	M	Q	Me	C	20	H	Q	Me	Mod
3	L	Q	Hi	UC	12	M	Q	Hi	Mod	21	H	Q	Hi	UC
4	L	N	Lo	VUC	13	M	N	Lo	UC	22	H	N	Lo	VUC
5	L	N	Me	UC	14	M	N	Me	Mod	23	H	N	Me	UC
6	L	N	Hi	VUC	15	M	N	Hi	UC	24	H	N	Hi	VUC
7	L	VN	Lo	EUC	16	M	VN	Lo	VUC	25	H	VN	Lo	EUC
8	L	VN	Me	VUC	17	M	VN	Me	UC	26	H	VN	Me	VUC
9	L	VN	Hi	EUC	18	M	VN	Hi	VUC	27	H	VN	Hi	EUC

**Table 2:** Fuzzy Rule Base of FLC2.

No.	WC	RC	VS	WRS	No.	WC	RC	VS	WRS	No.	WC	RC	VS	WRS
1	VB	VBa	Sl	CD	10	B	VBa	Sl	MD	19	G	VBa	Sl	N/MD
2	VB	VBa	Mo	VHD	11	B	VBa	Mo	HD	20	G	VBa	Mo	MD
3	VB	VBa	Fa	VHD	12	B	VBa	Fa	VHD	21	G	VBa	Fa	HD
4	VB	Ba	Sl	CD	13	B	Ba	Sl	MD	22	G	Ba	Sl	N/MD
5	VB	Ba	Mo	CD	14	B	Ba	Mo	CD	23	G	Ba	Mo	MD
6	VB	Ba	Fa	VHD	15	B	Ba	Fa	HD	24	G	Ba	Fa	CD
7	VB	Go	Sl	MD	16	B	Go	Sl	N/MD	25	G	Go	Sl	N/MD
8	VB	Go	Mo	CD	17	B	Go	Mo	MD	26	G	Go	Mo	N/MD
9	VB	Go	Fa	HD	18	B	Go	Fa	CD	27	G	Go	Fa	MD

**Table 3:** Fuzzy Rule Base of FLC3.

No.	RR	BTV	HR	DVS	No.	RR	BTV	HR	DVS	No.	RR	BTV	HR	DVS
1	Sl	Sm	S	B	10	Nm	Sm	S	F	19	Fs	Sm	S	B
2	Sl	Sm	No	F	11	Nm	Sm	No	G	20	Fs	Sm	No	F
3	Sl	Sm	Fa	B	12	Nm	Sm	Fa	F	21	Fs	Sm	Fa	B
4	Sl	H	S	VB	13	Nm	H	S	VB	22	Fs	H	S	VB
5	Sl	H	No	B	14	Nm	H	No	F	23	Fs	H	No	B
6	Sl	H	Fa	VB	15	Nm	H	Fa	VB	24	Fs	H	Fa	VB
7	Sl	VH	S	EB	16	Nm	VH	S	EB	25	Fs	VH	S	EB
8	Sl	VH	No	VB	17	Nm	VH	No	VB	26	Fs	VH	No	VB
9	Sl	VH	Fa	EB	18	Nm	VH	Fa	EB	27	Fs	VH	Fa	EB

**Table 4:** Fuzzy Rule Base of FLC4.

No.	VIE	WRS	DVS	DRM	No.	VIE	WRS	DVS	DRM	No.	VIE	WRS	DVS	DRM
1	EUC	N/MD	EB	Sv	43	VUC	HD	B	Sv	85	Mod	MD	G	VL
2	EUC	N/MD	VB	VH	44	VUC	HD	F	VH	86	Mod	CD	EB	Hg
3	EUC	N/MD	B	Hg	45	VUC	HD	G	Hg	87	Mod	CD	VB	Hg
4	EUC	N/MD	F	Co	46	VUC	VHD	EB	D	88	Mod	CD	B	Co
5	EUC	N/MD	G	Md	47	VUC	VHD	VB	D	89	Mod	CD	F	Md
6	EUC	MD	EB	D	48	VUC	VHD	B	D	90	Mod	CD	G	Lw
7	EUC	MD	VB	Sv	49	VUC	VHD	F	Sv	91	Mod	HD	EB	VH
8	EUC	MD	B	VH	50	VUC	VHD	G	VH	92	Mod	HD	VB	VH
9	EUC	MD	F	Hg	51	UC	N/MD	EB	Hg	93	Mod	HD	B	Hg
10	EUC	MD	G	Co	52	UC	N/MD	VB	Co	94	Mod	HD	F	Co
11	EUC	CD	EB	D	53	UC	N/MD	B	Md	95	Mod	HD	G	Md
12	EUC	CD	VB	D	54	UC	N/MD	F	Lw	96	Mod	VHD	EB	Sv
13	EUC	CD	B	Sv	55	UC	N/MD	G	VL	97	Mod	VHD	VB	Sv
14	EUC	CD	F	VH	56	UC	MD	EB	VH	98	Mod	VHD	B	VH
15	EUC	CD	G	Hg	57	UC	MD	VB	Hg	99	Mod	VHD	F	Hg
16	EUC	HD	EB	D	58	UC	MD	B	Co	100	Mod	VHD	G	Co
17	EUC	HD	VB	D	59	UC	MD	F	Md	101	C	N/MD	EB	Md
18	EUC	HD	B	Sv	60	UC	MD	G	Lw	102	C	N/MD	VB	Lw
19	EUC	HD	F	Sv	61	UC	CD	EB	Sv	103	C	N/MD	B	VL
20	EUC	HD	G	VH	62	UC	CD	VB	VH	104	C	N/MD	F	Sf
21	EUC	VHD	EB	D	63	UC	CD	B	Hg	105	C	N/MD	G	Sf
22	EUC	VHD	VB	D	64	UC	CD	F	Co	106	C	MD	EB	Co
23	EUC	VHD	B	D	65	UC	CD	G	Md	107	C	MD	VB	Md
24	EUC	VHD	F	D	66	UC	HD	EB	D	108	C	MD	B	Lw
25	EUC	VHD	G	Sv	67	UC	HD	VB	Sv	109	C	MD	F	VL
26	VUC	N/MD	EB	VH	68	UC	HD	B	VH	110	C	MD	G	VL
27	VUC	N/MD	VB	Hg	69	UC	HD	F	Hg	111	C	CD	EB	Hg
28	VUC	N/MD	B	Co	70	UC	HD	G	Co	112	C	CD	VB	Co
29	VUC	N/MD	F	Md	71	UC	VHD	EB	D	113	C	CD	B	Md
30	VUC	N/MD	G	Lw	72	UC	VHD	VB	D	114	C	CD	F	Lw
31	VUC	MD	EB	Sv	73	UC	VHD	B	Sv	115	C	CD	G	Lw
32	VUC	MD	VB	VH	74	UC	VHD	F	VH	116	C	HD	EB	VH
33	VUC	MD	B	Hg	75	UC	VHD	G	Hg	117	C	HD	VB	Hg
34	VUC	MD	F	Co	76	Mod	N/MD	EB	Md	118	C	HD	B	Co
35	VUC	MD	G	Md	77	Mod	N/MD	VB	Md	119	C	HD	F	Md
36	VUC	CD	EB	D	78	Mod	N/MD	B	Lw	120	C	HD	G	Md
37	VUC	CD	VB	Sv	79	Mod	N/MD	F	VL	121	C	VHD	EB	Sv
38	VUC	CD	B	VH	80	Mod	N/MD	G	Sf	122	C	VHD	VB	VH
39	VUC	CD	F	Hg	81	Mod	MD	EB	Co	123	C	VHD	B	Hg
40	VUC	CD	G	Co	82	Mod	MD	VB	Co	124	C	VHD	F	Co
41	VUC	HD	EB	D	83	Mod	MD	B	Md	125	C	VHD	G	Co
42	VUC	HD	VB	D	84	Mod	MD	F	Lw					
